# Modulation of Lipopolysaccharide Stimulated Nuclear Factor kappa B Mediated iNOS/NO Production by Bromelain in Rat Primary Microglial Cells

**DOI:** 10.7508/ibj.2016.01.005

**Published:** 2016-01

**Authors:** Soraya Abbasi Habashi, Farzaneh Sabouni, Ali Moghimi, Saeed Ansari Majd

**Affiliations:** 1 Center for Neuroscience and Behavior, Department of Biology, Faculty of Science, Ferdowsi University of Mashhad, Mashhad, Iran;; 2 National Institute of Genetic Engineering and Biotechnology, Tehran, Iran

## Abstract

**Background:**

Microglial cells act as the sentinel of the central nervous system .They are involved in neuroprotection but are highly implicated in neurodegeneration of the aging brain. When over-activated, microglia release pro-inflammatory factors, such as nitric oxide (NO) and cytokines, which are critical in eliciting neuroinflammatory responses associated with neurodegenerative diseases. This study examined whether bromelain, the pineapple-derived extract, may exert an anti-inflammatory effect in primary microglia and may be neuroprotective by regulating microglial activation.

**Methods:**

Following the isolation of neonatal rat primary microglial cells, the activation profile of microglia was investigated by studying the effects of bromelain (5, 10, 20, and 30 µg/ml) on the levels of NO, inducible nitric oxide synthase (iNOS), and nuclear factor kappa B (NF-κB) in microglia treated with lipopolysaccharide (LPS) (1 µg/ml). Data were analyzed using Student's t-test. *P* values less than 0.05 were considered to be statistically significant, compared with the LPS-treated group without bromelain.

**Results:**

Results showed that pretreatment of rat primary microglia with bromelain, decreased the production of NO induced by LPS (1 µg/ml) treatment in a dose-dependent manner. Bromelain (30 µg/ml) also significantly reduced the expression of iNOS at mRNA level and NF-κB at protein level. Moreover, the study of mitochondrial activity in microglia indicated that bromelain had no cytotoxicity at any of the applied doses, suggesting that the anti-inflammatory effects of bromelain are not due to cell death.

**Conclusion:**

Bromelain can be of potential use as an agent for alleviation of symptoms in neurodegenerative diseases.

## INTRODUCTION

Microglia are the innate immune cells or resident macrophages that can exist in activated and inactivated forms and are believed to regulate inflammatory responses in the central nervous system )CNS(^[^^1^^,^^2^^]^. In response to a plethora of stimuli, microglia become activated and then initiate an inflammatory cascade in the CNS that contributes to the pathogenesis of Alzheimer’s disease, Parkinson’s disease, multiple sclerosis, AIDS dementia complex, and ischemia^[^^3^^-^^5^^]^. Activated microglia have been shown to produce inflammatory mediators including NO and cytokines such as the tumor necrosis factor-α^[^^6^^,^^7^^]^.

Although microglia play a protective role by releasing trophic factors and removing dead cells, chronic microglial activation and consequent over-production of pro-inflammatory mediators lead to the initiation and progression of several neurodegenerative diseases^[^^8^^-^^10^^]^. 

A number of anti-inflammatory reagents can prevent microglial activation or microglial production of inflammatory cytokines in CNS disease conditions and attenuate neuronal degeneration^[^^11^^,^^12^^]^. Thus, the efficient control of microglial activation in numerous neuro-degenerative diseases is regarded as a main therapeutic approach.

Bromelain is a reagent derived from the pineapple stem (*Ananas comosus*), which is known for its anti-inflammatory effects^[^^13^^,^^14^^]^. Numerous pharmacological activities associated with bromelain have been reported that include the regulation of immune functions, anti-inflammation, anti-edema, anti-hypertension, reduction of thrombogenesis, and inhibition of cancer cell growth^[^^15^^-^^18^^]^. Bromelain has been shown to enhance IFN-γ-mediated NO and tumor necrosis factor-α production by macrophages and also increases IFN-γ production by natural killer cells following the activation with IL-2 and IL-12. On the other hand, cross-linked bromelain has been reported to reduce LPS-induced nuclear factor kappa B (NF-κB) activity and cyclooxygenase-2 mRNA as well as prostaglandin E2 expression in BV-2 microglial cells^[^^19^^-^^21^^]^. 

Although there are some controversial effects of bromelain, its efficacy in primary microglial cells activation has not been reported yet^[^^20^^-^^22^^]^. Hence, this study examined whether bromelain represses micro-glial activation, thereby conferring neuroprotection against inflammation-related neuronal injury. 

## MATERIALS AND METHODS

**Materials and reagents**

DMEM containing L-arginine (200 mg/l), FBS, other tissue culture reagents, and Griess reagent were purchased from Gibco BRL (Grand Island, NY, USA). LPS (E5:055), bromelain, proteinase inhibitor E-64, 3-(4,5-dimethylthiazol-2-yl)-2,5-diphenyltetra-zolium bromide (MTT), and bovine serum albumin were purchased from Sigma Chemical Co. (St. Louis, MO, USA). Horse radish peroxidase-conjugated anti-mouse IgG and anti-goat IgG were obtained from Invitrogen (Carlsbad, USA) and OX-42 antibodies from Boehringer Mannheim (Indianapolis, IN, USA). The RNA isolation kit was purchased from Intron Biotechnology (Korea), and all the reagents and enzymes for RT-PCR were from Fermentas (Vilnius, Lithuania). The p65 antibodies and the antibody against ß-actin were supplied by Santa Cruz Biotechnology (Santa Cruz, CA, USA) and Sigma, respectively.

**Inhibition of the proteolytic activity of bromelain**

Diluted bromelain (10 mg/ml) in 3 µM dithiothreitol was incubated with 100 µM E-64 and 60 mM sodium acetate (pH 5) at 37^o^C for 10 min. The inactivated bromelain was then dialyzed in PBS at 4^o^C overnight. The total inactivation of bromelain was achieved as assayed with casein^[^^23^^]^.

**Cell culture**

Primary microglial cells were prepared from cerebral cortices of one-day-old rat pups as described previously^[^^24^^]^. Briefly, the cells were cultured in DMEM, supplemented with 100 UI/ml penicillin G, 100 μg/ml streptomycin, 2 mmol/L L-glutamine, 0.011 g/L pyruvate, and 10% fetal calf serum. The cells were then seeded on polystyrene culture dishes (Nunc, USA) and incubated in a humidified atmosphere containing 5% CO_2_ at 37°C for two days. To obtain primary microglia-rich mixed glial cultures, all media and tissues were removed, and fresh media were replaced after two days.

**Isolation of microglia**

After the cells became confluent at 12–14 days, the flasks were shaken to remove the microglia^[^^25^^]^ and re-plated at 1×10^5 ^in a 96-well tissue culture. The detached microglial cells were incubated for one hour, and the non-adherent cells were removed. The adherent microglial cells were cultured for 24 h, and the purity of the cultures was routinely found to be greater than 95%, as judged by immunostaining with an anti-OX-42 antibody. 

**Bromelain treatment**

Primary microglial cells were pretreated with bromelain (5, 10, 20, and 30 µg/ml) in a fresh medium containing 1% FBS for one hour before LPS (1 µg/ml) addition. The cells were then incubated for 48 h.

**Cell viability assay**

After various treatments, the medium was removed, and the cells were incubated with MTT solution (1 mg/ml) in a culture medium at 37ºC for 4 h. The MTT solution was then removed, the formazan crystals in cells were dissolved in dimethyl sulfoxide, and MTT formazan levels were determined by measuring the absorbance at 580 nm using a microplate reader.

**Nitrite assay**

NO production was assessed as nitrite (NO_2_^-^) accumulation in the culture medium of three independent experiments 48 h after treatment using a colorimetric test based on Griess reagent^[^^26^^]^. Sodium nitrite, NaNO_2_, standard titration (0-150 µM) solution (diluted with water prior to use) was used for construction of the standard curve.

**Reverse transcription polymerase chain reaction**

Total RNA was isolated from the microglial cells 48 h after LPS stimulation using the Easy-Blue^TM^ Total RNA Extraction Kit as instructed by the manufacturer. For RT-PCR, a 2-µg sample of total RNA was reverse transcribed according to the manufacturer's instructions (Pure Extreme, Fermentas, USA). Single stranded cDNA was amplified by PCR with primers for **inducible NO synthase** (iNOS) and glyceraldhyde-3-phosphate dehydrogenase, whose primer sequences are shown in Table 1. The cycle profile used was performed in an initial denaturation at 95°C for 1 min, followed by 35 cycles at 95°C for 30 s, 59°C for 30 s, and 72°C for 30 s. The glyceraldhyde-3-phosphate dehydrogenase was used as the internal control to evaluate the relative expression of iNOS. Densitometry analysis of the bands was performed by Totallab software, version 1.10.

**Western-blotting**

Forty hours after LPS stimulation, the cells were washed three times in PBS and lysed with lysis buffer [1% Triton X-100, 50 mM Tris–Cl, 150 mM NaCl, and 1 mM phenyl methyl sulfonyl fluoride]. Equal amounts of protein (10 µg) were separated electrophoretically using 10% sodium dodecyl sulfate polyacrylamide gel electrophoresis. Subsequently, the gel was transferred to a 0.45-µm polyvinylidene fluoride transfer membrane (Millipore, USA). The membrane was soaked in a blocking buffer (5% skimmed milk), incubated with primary antibodies (anti-p65, 1:200; anti-ß-Actin, 1:25000) overnight, followed by goat anti-rabbit horse radish peroxidase conjugate antibodies (1:10000). The immune complexes were then visualized using an ECL chemiluminescence system. Densitometry analysis of the bands was performed by Totallab software.

**Statistical analysis**

Data were presented as the mean ± S.E.M. of at least three separate experiments. Comparisons between two groups were analyzed using student's *t*-test. *P* values less than 0.05 were considered to be statistically significant, compared with the LPS-treated group without bromelain.

## RESULTS

**Effects of bromelain on NO production in LPS-stimulated primary microglia**

Potential anti-inflammatory activity of bromelain in primary microglia was tested by evaluating the production of the inflammatory mediator, NO, in the culture media using the Griess assay. Rat primary microglial cultures were pretreated with bromelain (5, 10, 20, and 30 µg/ml) for one hour prior to stimulation with LPS (1 µg/ml), which subsequently involved a 48-h incubation period. 

**Fig. 1:**
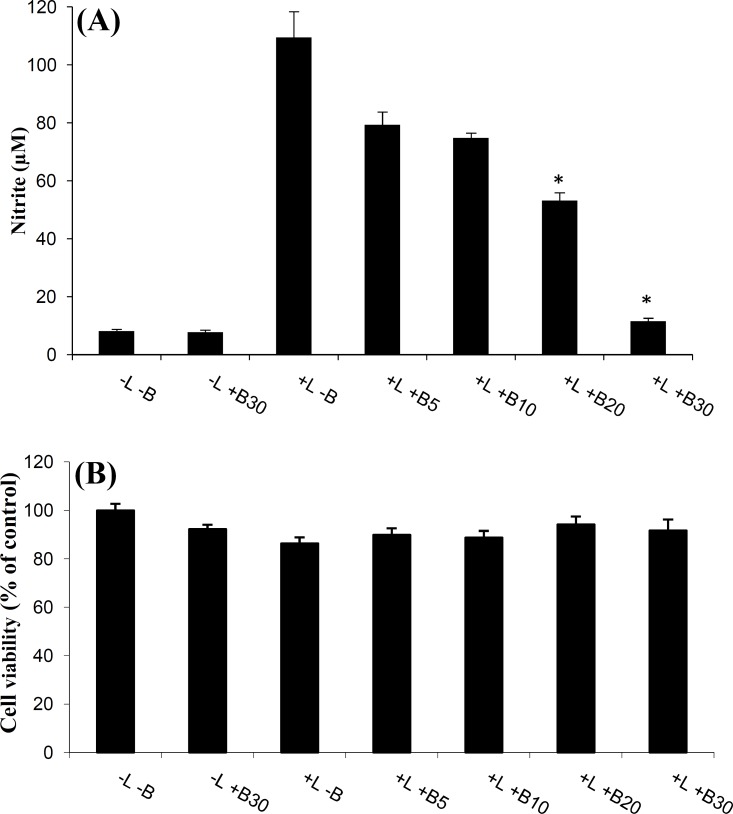
The effects of bromelain on NO production in LPS-stimulated microglial cells. Primary microglial cells were incubated in the absence (-) or presence (+) of 1 µg/ml LPS. The cells were pretreated with various amounts of bromelain (5, 10, 20, and 30 µg/ml) for one hour prior to LPS addition. Following 48 h of incubation, the cultures were subjected to a nitrite assay (A) and a cell viability assay (B). The LPS-stimulated microglial cells showed a remarkable increase in NO levels in the cell-conditioned media when compared to those in the control. Pretreatment of microglial cells with bromelain (at 5-30 µg/ml) significantly reduced NO production in the LPS-stimulated primary microglia in a dose-dependent manner. The MTT assay indicates that the inhibitory effects of bromelain on LPS-stimulated NO production are not due to cytotoxic action of bromelain on primary microglia. *P<0.01 as compared with the LPS-treated group without bromelain

The LPS-stimulated microglial cells showed a remarkable increase in NO levels in the cell-conditioned media when compared to those in the control. Pretreatment of microglial cells with bromelain (at 5-30 µg/ml) significantly reduced NO production in the LPS-stimulated primary microglia in a dose-dependent manner (Fig. 1A).

**Assessment of bromelain toxicity**

In order to investigate the cytotoxic action of bromelain during the inhibition of LPS-stimulated NO production, the effect of bromelain on cell viability was evaluated. The MTT assays showed that there was no significant reduction in cell viability (Fig. 1B), which indicates that the inhibitory effects of bromelain on LPS-stimulated NO production is not due to cytotoxic action of bromelain on primary microglia. 

**Suppression of LPS-induced iNOS mRNA expression in primary microglial cells**

**Fig. 2.**
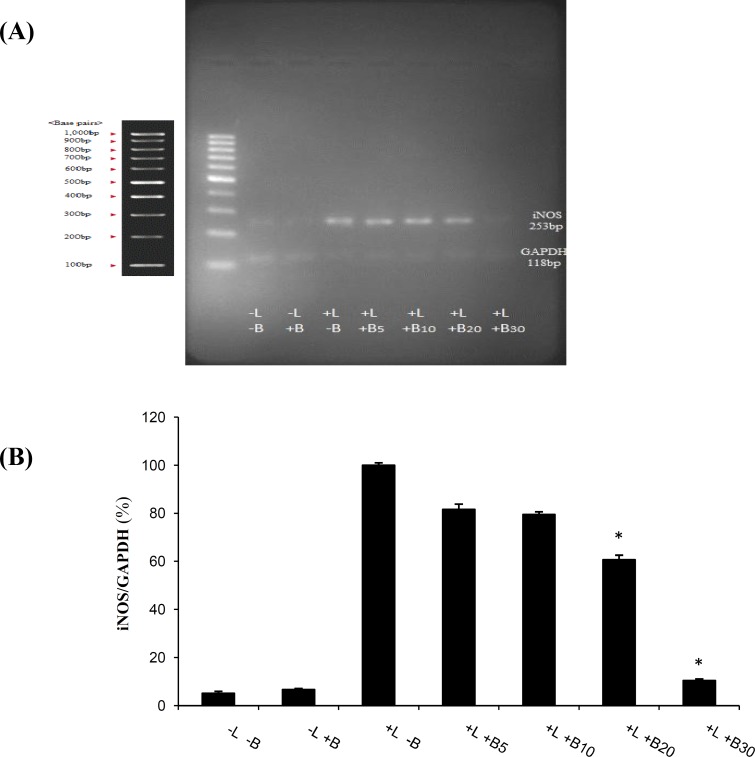
Effects of bromelain on the levels of iNOS production by LPS-stimulated primary microglial cells. Primary microglial cells were pretreated with bromelain at 5-30 µg/ml for one hour before LPS (1 µg/ml) addition. (A) After incubation for 48 hours, the expression of iNOS mRNA levels was measured by semi-quantitative RT-PCR analysis. The results showed that the LPS-stimulated increase of iNOS levels in the primary microglial cells was reduced to the control levels in the presence of 30 µg/ml bromelain. LPS and bromelain treatments are shown with L and B, respectively in the Figure. (B) Densitometry analysis of the bands was performed by Totallab software. *P<0.01 as compared with the LPS-treated group without bromelain

To determine whether the reduction in LPS-induced NO levels, as measured in supernatants, was associated with the decrease in the steady-state iNOS (the enzyme responsible for the production of NO) mRNA levels, RT-PCR analyses were conducted. Microglial cells were pretreated with bromelain (5-30 µg/ml) for one hour and stimulated with LPS (1 µg/ml). As anticipated, LPS markedly increased iNOS mRNA expression in rat primary microglia, but pretreatment with bromelain at 30 µg/ml significantly downregulated iNOS mRNA expression (Fig. 2).

**Table 1:** RT-PCR primers

**Gene**	**Primer Pair (5΄-3΄)**	**Size (bp)**	**GenBank number**
*GAPDH*	F: CCCCCAATGTATCCGTTGTG R: TAGCCCAGGATGCCCTTTAGT	118	BC059110
			
*iNOS*	F: GACATCGACCAGAAGCTGTC R: GGGCTCTGTTGAGGTCTAAAG	253	MMU43428

GAPDH, glyceraldhyde-3-phosphate dehydrogenase; F, forward; R, reverse

**Inhibition of LPS-induced NF-κB protein expression in primary microglial cells**

**Fig. 3:**
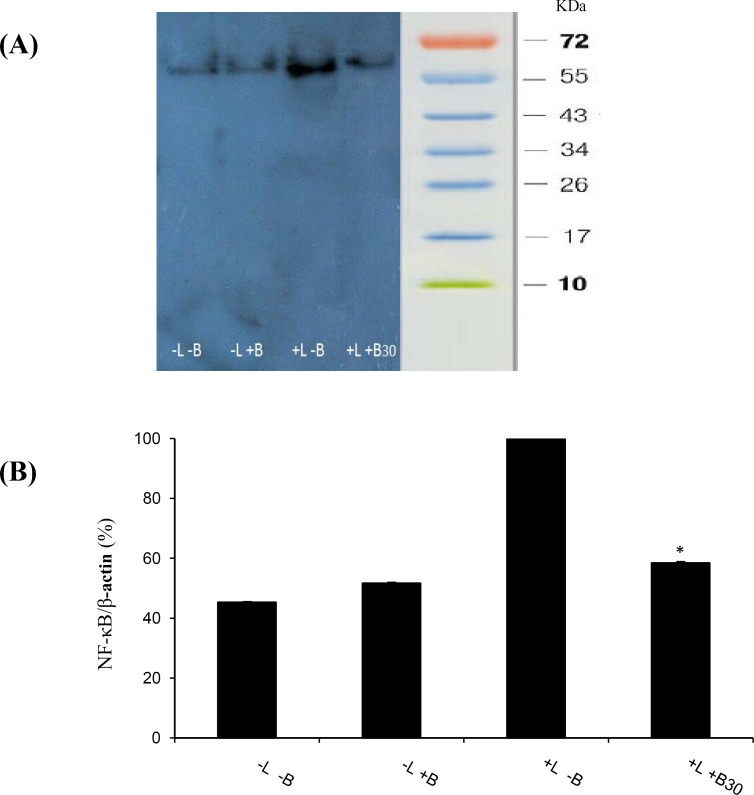
Effects of bromelain on the levels of NF-κB p65 production by LPS-stimulated primary microglial cells. Primary microglial cells were pretreated with bromelain at 30 µg/ml for one hour before LPS (1 µg/ml) addition. (A) After incubation for 48 hours, the expression of NF-κB p65 protein levels was measured by semi-quantitative Western-blot analysis. The results showed that the LPS-stimulated increase of NF-κB levels in the primary microglial cells was reduced to the control levels in the presence of 30 µg/ml bromelain. LPS and bromelain treatments are shown with L and B respectively in the picture. (B) Densitometry analysis of the bands was performed by Totallab. *P<0.01 as compared with the LPS-treated group without bromelain

To further confirm the involvement of the NF-κB pathway, we investigated the effects of bromelain at 30 µg/ml on LPS-induced p65 expression in microglial cells. As shown in Figure 3, LPS (1 µg/ml) treatment significantly increased NF-κB expression in the primary cultured microglia, which was assessed by the measurement of p65 protein expression. Western-blot analysis showed that the LPS-stimulated increase of NF-κB levels in the primary microglial cells was reduced to the control levels in the presence of 30 µg/ml bromelain (Fig. 3). The results were confirmed by standardized values (NF-κB/internal standard protein, β-actin). 

## DISCUSSION

 Activation of microglia has both beneﬁcial and harmful effects on neuronal injury in neurodegenerative diseases^[^^25^^,^^27^^]^. Over-activation of microglia contributes to neurodegenerative processes through the production of various neurotoxic factors including NO^28^. Many researchers have shown that NO produc-tion is up-regulated in the activated microglia^[^^29^^,^^30^^]^.

Thus, it is suggested that the search for efficient anti-inﬂammatory compounds that attenuate microglial activation may lead to an effective therapeutic approach against many neurodegenerative conditions. This study demonstrated that the bromelain was capable of decreasing the inﬂammatory activation of microglia in culture. Furthermore, the levels of the pro-inflammatory factor, NO, were evaluated in activated primary microglia treated with bromelain. 

Among the various biological activities of bromelain, its anti-inflammatory efficacy has created interest in its meshanisms of action. However, there are inconsistencies regarding the effects of bromelain on inflammation. It has been shown that bromelain can simultaneously stimulate and inhibit immune cell responses *in vitro* and *in vivo*^[^^19^^,^^21^^]^. At a concentration of 50 µg/ml, bromelain has been reported to increase the production of IFN-γ-stimulated nitrite in murine macrophage cell lines^[19]^, whereas in other reports, the concentration of 100 µg/ml has been found to significantly inhibit the enhanced production of LPS-induced nitrite in the same cell lines^[^^31^^]^. In addition, researchers have shown that bromelain can have anti-inflammatory effects on LPS-activated microglial cell lines^[^^21^^]^.

In fact, our study demonstrated that bromelain (at 5-30 µg/ml) could reduce LPS-stimulated NO production in the rat primary microglial cells (Fig. 1). It has been also indicated that the potent anti-inflammatory effect of bromelain, due to a decreased production of NO, is dose dependent at 10, 20, and 30 µg/ml. By considering the results of previous researches and those of the present study, it is evident that the different effects of bromelain, which have been observed so far, are more likely due to cell type and dosage. Moreover, the presence of viable cells indicates the lack of cytotoxicity of bromelain at any of the applied doses, suggesting that the anti-inflammatory effects of bromelain are not due to cell death.

The present study showed that bromelain at the concentration of 30 µg/ml significantly inhibited LPS (1 µg/ml)-induced nitrite overproduction in the rat primary microglial cells, suggesting that bromelain decreases iNOS activity.

The results have suggested that the modulation of iNOS gene expression by bromelain is involved in the improvement of the increased activation of microglial cells. Furthermore, treatment by bromelain has been reported to inhibit over-expressed iNOS mRNA and LPS-induced nitrite over-production in macrophage cell lines^[^^31^^]^. Confirming Wen's results^[^^32^^]^, our study showed that bromelain treatment (30 µg/ml) suppresses the activation of primary microglia accompanied by a significant reduction in the overexpression of microglial iNOS mRNA (Fig. 2).

NF-κB is an important modulator of iNOS expression in microglia. It is also an important target of several anti-inflammatory drugs^[^^32^^,^^33^^]^. According to our results, bromelain treatment normalized up-regulated iNOS mRNA and inhibited LPS-induced NF-κB expression in rat primary microglial cells. The results suggest that the reduction of the overexpressed iNOS mRNA in the primary microglia is probably through the modulation of the NF-κB pathway. 

Chemical components derived from natural sources have attracted much attention in the area of health and disease. The inhibition of microglial inflammatory responses is considered a promising target for the treatment of many neuropathologies through control-ing microglial activation^[^^33^^]^. The role of bromelain in reducing the pro-inﬂammatory mediators, NO, iNOS, and NF-κB suggests that bromelain can be used as a useful anti-inflammatory agent.
